# An evaluation system for postgraduate pediatric residency programs: report of a 3-year experience

**DOI:** 10.1007/s00431-017-2967-z

**Published:** 2017-08-01

**Authors:** Liviana Da Dalt, Pasquale Anselmi, Sara Furlan, Silvia Carraro, Eugenio Baraldi, Egidio Robusto, Giorgio Perilongo

**Affiliations:** 1 Pediatric Residency Program, Department of Woman’s and Child’s Health, University of Padua, Via Giustiniani 3, 35128 Padua, Italy; 2 Department of Philosophy, Sociology, Educational Studies and Applied Psychology, University of Padua, Padua, Italy

**Keywords:** Pediatrics, Academic training, Assessment, educational, Medical residency

## Abstract

The way a postgraduate medical training program is organized and the capacity of faculty members to function as tutors and to organize effective professional experiences are among the elements that affect the quality of training. An evaluation system designed to target these elements has been implemented within the framework of the Pediatric Residency Program of the University of Padua (Italy). The aim of this report is to describe some aspects of the experience gained in the first 3 years of implementation of the system (2013–2015). Data were collected using four validated questionnaires: the “Resident Assessment Questionnaire”, the “Tutor-Assessment Questionnaire”, the “Rotation-Assessment Questionnaire”, and the “Resident Affairs Committee-Assessment Questionnaire”. The response rate was 72% for the “Resident Assessment Questionnaires”; 78% for the “Tutor-/Rotation-Assessment Questionnaires” and 84% for the “Resident Affair Committee-Assessment Questionnaires”. The scores collected were validated by psychometric tests.

*Conclusion*: The high rates of completed questionnaires returned and the psychometric validation of the results collected indicate that the evaluation system reported herein can be effectively implemented. Efforts should be made to refine this system and, more importantly, to document its impact in improving the Pediatric Residency Program.

**Table:** 

**What is known:** *• The elements that influence the quality of postgraduate training programs and the knowledge, performance, and competences of residents must be regularly assessed.* *• Comprehensive evaluation systems for postgraduate residency programs are not universally implemented also because quite often common guidelines and rules, well-equipped infrastructures, and financial resources are missing*.
**What is new:** • *We show the feasibility of implementing an evaluation system that targets some of the key elements of a postgraduate medical training program in Italy, a European country in which the regulations governing training programs and, notably, the evaluation of residents are still being developed*.

## Introduction

Postgraduate medical training programs should be continuously monitored to guarantee the quality of the education curriculum [4]. The way the program is run and the capacity of the faculty members to function as tutors and to organize effective professional experiences are elements which, among others, can affect the learning experience of residents and, ultimately, the quality of doctors licensed to practice medicine. The Pediatric Residency Program of the University of Padua (Italy) has adopted an evaluation system specifically designed to target these elements. This report describes the experience gained over the first 3 years (2013–2015).

## Materials and methods

The Pediatric Residency Program of the University of Padua is a Ministerial accredited and ISO-9001 certified 5-year program that offers a Diploma of Specialization in Pediatrics [1]. An average of 90 residents attends the program every year. Approximately, 80% of the learning activities takes place in the clinical setting under faculty supervision. During their first 3 years, residents rotate through an average of 12 Pediatric Divisions of the Department of Woman’s and Child’s Health of the University Hospital of Padua. These rotations range from 3 to 6 months. During the last 2 years, residents select elective rotations involving at the most two divisions, each lasting from 9 to 15 months. Each Division Chief is responsible for organizing the rotation, for being tutor for residents and for evaluating them. The Resident Affairs Committee is the body in charge of running the program.

To monitor the program, the Resident Affairs Committee of the Pediatric Residency Program has developed an evaluation system according to which Division Chiefs (tutors) are requested to evaluate residents, and residents are requested to evaluate the quality of the training provided by each Division Chief, the professional experience acquired during the various rotations, and the functioning of the Resident Affairs Committee.

This evaluation system is based on four validated questionnaires: the “Residents Assessment Questionnaire (AQ)”, the “Tutor-AQ”, the “Rotation-AQ” and the “Resident Affairs Committee-AQ” [2, 3] that consist of various items evaluated on a four-point rating scale (from 1 “poor” to 4 “excellent”). The “Resident-AQs” are completed by the Division Chiefs, who, as mentioned above, are also the “tutors” that the residents evaluate by completing the “Tutor-AQ” questionnaire. The “Resident-, Tutor- and Rotation-AQs should be completed within two weeks from the end of each rotation, while the “Resident Affairs Committee-AQs” should be completed and returned at the end of each academic year. The “Tutor-AQ” is anonymous. The results of these questionnaires, except “Resident Affairs Committee-AQs”, are communicated by the Resident Affair Committee to the residents and tutors during individual private meetings. Once a year, the average scores obtained during a 12-month period by each tutor and rotation are presented to the whole faculty for discussion. Only residents of the first 4 years undergo regular evaluation (“formative assessment”) because the results of the “Resident-AQs” are meant to provide information to adjust their training program, if necessary. Fifth-year residents receive only a single final evaluation (“summative evaluation”) regarding their one year-long conclusive elective. Consequently, they are not required to complete either the “Tutor-AQ” or the “Rotation-AQ”.

An internet-based system has been developed to handle the entire evaluation process. The web-based system provides the Resident Affairs Committee with a dashboard command to monitor the implementation and the correct use of the evaluation system. On the whole, questionnaires can be completed in less than 10 min. Tutorials to instruct new residents and faculty members on how to complete the questionnaires are organized annually. The psychometric properties of the questionnaires, although previously validated, are regularly monitored to verify (a) the internal consistency of all the questionnaires, namely, to verify whether differences among the scores are true and not due to measurement errors [5]; (b) the strength of the items in defining a unidimensional measure [7]; and (c) the level of agreement among respondents, namely, that the tutors substantially agree in their evaluations of the residents, and that the residents agree in their evaluations of tutors, rotations and Resident Affairs Committee [6, 8]. At the end of the first 3 years of implementation of the evaluation system, students and faculty were asked to provide feedbacks about the system in terms of (a) purposes, (b) comprehensiveness, and (c) user-friendliness. These items are evaluated on a four-point rating scale, from 1 “poor” to 4 “excellent”.

## Results

### The results of the questionnaires

The number of questionnaires expected and collected in the study period is reported in Table 1.

**Table 1:** Number of questionnaires collected in the years 2013, 2014, and 2015

Questionnaire	Year	N. residents	Number of evaluations		
			Expected	Provided within 15 days	Provided
Resident-AQ	2013	71	227	95 (42%)	153 (67%)
	2014	71	237	143 (60%)	165 (69%))
	2015	54	158	104 (65%)	129 (82%)
Total			622	342 (55%)	447 (72%)
Tutor-and Rotation-AQ	2013	71	227	163 (73%)	185 (81%)
	2014	71	237	139 (59%)	170 (80%)
	2015	54	158	101 (64%)	130 (82%)
Total			622	403 (65%)	485 (78%)
RAC-AQ	2013	86	86	68 (79%)	71 (83%)
	2014	89	89	58 (65%)	75 (84%)
	2015	73	73	60 (82%)	63 (86%)
Total			248	186 (75%)	209 (84%)

*AQ* Assessment Questionnaire, *RAC* Resident Affair Committee

#### Resident-AQ

Tutors returned 447 “Resident-AQs”, which represented 72% of the total ones which were supposed to be returned; 55% were returned within the 15-days requested. The number of “Resident-AQs” received per resident in a year varied from one (for fourth year residents) to four (for the ones attending the first 3 years).

#### Tutor-AQ and Rotation-AQ

Four-hundred and eighty-five “Tutor-AQs” and “Rotation-AQs” were returned (i.e., 78% of those expected) of which 65% were returned with the 15-day deadline.

#### Resident Affairs Committee-AQ

Two-hundred and nine “Residents Affairs Committee-AQs” were returned (i.e., 84% of those expected; 75% of them were returned within 15 days. Ninety-nine residents evaluated the Resident Affairs Committee from one to three times.

**Fig. 1:**
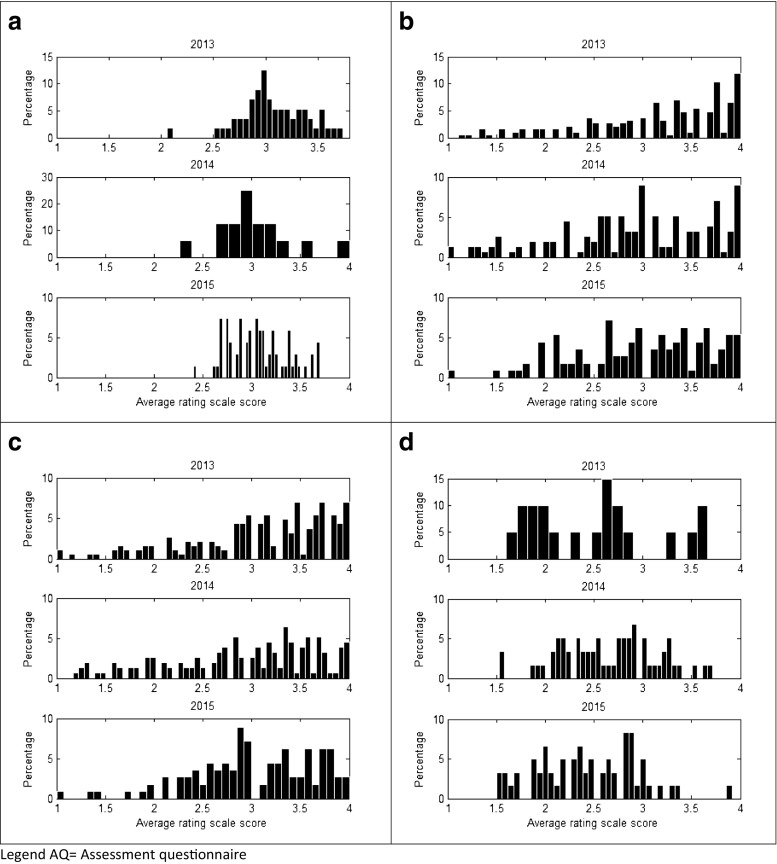
Percentage of average rating scale scores given to the Resident-AQ (**a**), Tutor-AQ (**b**), Rotation-AQ (**c**), and Resident Affair Committee-AQ (**d**), for the years 2013, 2014, and 2015

Tutors tended to be generous in assessing the performance of residents (Fig. 1a), while residents in judging tutors, the professional value of the rotations and the Resident Affairs Committee used more often the entire scale of scores (Fig. 1b–d).

**Fig. 2:**
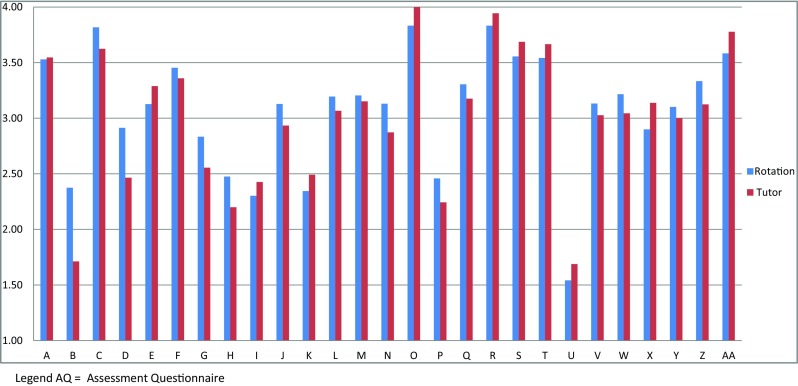
An example of the reports of the combined “Tutor-AQs” and “Rotation-AQs” that were presented during the yearly general faculty meeting showing the average scores assigned to each tutor (Division Chief) and to each rotation, in a 12-month period (median score from 0 to 4). Each *letter* represents a single “rotation” and its own “tutor”

Figure 2 shows an example of the reports of the combined “Tutor-AQs” and “Rotation-AQs” that were presented during the yearly general faculty meeting: the average scores assigned to each tutor (Division Chief) and to each rotation in a year are shown.

The psychometric properties of the questionnaires (as outlined in the “Methods”) were invariably confirmed.

### Feedback of students and tutors regarding the evaluation system

Feedbacks on the system were received from 76% of residents and 58% of tutors. Interestingly, 80% of the tutors but only 60% of the residents felt that the purpose of the evaluation system was clear, that the meaning of the different items was easy to understand and that the entire system would help to improve the performance of residents and the quality of training. Most residents and tutors thought that the internet-based system was easy to use.

## Discussion

The relatively high rates of questionnaires completed and returned in time indicate that the evaluation system adopted within the framework of the University of Padua’s Pediatric Residency Program was successfully implemented. In fact, the analysis of the validity of the various responses provided, as assessed by conventional psychometric tests, and the fact that most tutors and residents returned the questionnaires within the time set, indicate that the task of completing the questionnaires was taken seriously by all.

The aim of the evaluation system was to provide residents, tutors and the Resident Affairs Committee with feedback regarding their performance, based on objective data, and thus to provide a base on which to continuously improve the program. We do not yet have objective data of the impact of this evaluation system on the quality of the Pediatric Residency Program. However, the presentation, during the yearly faculty meetings, of the scores obtained by each tutor and rotation has stimulated efforts to improve. Indeed, the scores assigned to tutors and rotations improved over the years. Furthermore, the fact that all the Division Chiefs were interested in having private discussions on the scores received is a sign that they took seriously the judgments received. The same applies to the residents, who were eager to receive, privately, feedback on their performance. Fortunately, no conflicts seemed to have emerged between residents and tutors and no one was offended by negative judgments. We believe that this evaluation system set a positive circle, considering the improvement of many scores of “Tutor-AQs” and “Rotation-AQs” documented over the years and the impression we had had that everybody took quite seriously the judgments received.

We acknowledge some intrinsic limitations of the system, namely, the fact that the “Tutor-AQ” was meant to target only the performance of the Division Chief, thus neglecting the contribution of all the other staff members. More should be done in order to refine it and, more importantly, to document its impact in improving the program.

Finally, we are aware that comprehensive evaluation systems require common guidelines and rules, well-equipped infrastructures and financial resources. However, we believe that good organization, strong motivation, and a culture of educational commitment can result in an excellent residency program, even with limited financial resources. This is the ultimate reason why we thought appropriate to share this limited experience.

## Abbreviations

AQ: Assessment Questionnaire
Resident Affair Committee-AQ: Resident Affair Committee-Assessment Questionnaire
Resident-AQ: Resident-Assessment Questionnaire
Rotation-AQ: Rotation-Assessment Questionnaire
Tutor-AQ: Tutor-Assessment Questionnaire
